# Genus-Wide Physicochemical Evidence of Extracellular Crystalline Silver Nanoparticles Biosynthesis by *Morganella* spp

**DOI:** 10.1371/journal.pone.0021401

**Published:** 2011-06-21

**Authors:** Rasesh Y. Parikh, Rajesh Ramanathan, Peter J. Coloe, Suresh K. Bhargava, Milind S. Patole, Yogesh S. Shouche, Vipul Bansal

**Affiliations:** 1 National Centre for Cell Science, Pune University Campus, Pune, India; 2 School of Applied Sciences, RMIT University, Melbourne, Victoria, Australia; Aristotle University of Thessaloniki, Greece

## Abstract

This study was performed to determine whether extracellular silver nanoparticles (AgNPs) production is a genus-wide phenotype associated with all the members of genus *Morganella*, or only *Morganella morganii* RP-42 isolate is able to synthesize extracellular Ag nanoparticles. To undertake this study, all the available *Morganella* isolates were exposed to Ag^+^ ions, and the obtained nanoproducts were thoroughly analyzed using physico-chemical characterization tools such as transmission electron microscopy (TEM), UV-visible spectrophotometry (UV-vis), and X-ray diffraction (XRD) analysis. It was identified that extracellular biosynthesis of crystalline silver nanoparticles is a unique biochemical character of all the members of genus *Morganella*, which was found independent of environmental changes. Significantly, the inability of other closely related members of the family Enterobacteriaceae towards AgNPs synthesis strongly suggests that AgNPs synthesis in the presence of Ag^+^ ions is a phenotypic character that is uniquely associated with genus *Morganella*.

## Introduction

As nanotechnology is emerging as an interdisciplinary field with potential to influence various aspects of human life through a myriad of applications, biological synthesis of nanomaterials is gaining particular attention as a rapidly growing discipline of Bio-nanotechnology with an enormous application potential in the coming future [1], [2]. There has been a strong interest in developing environmentally benign protocols for biological synthesis of nanomaterials that do not involve toxic chemicals in synthesis process. As demonstrated previously by our group and others [1]–[4], this has been successfully achieved by biological synthesis of various metal (Au [5]–[7], Ag [8]–[12], and Pt [13]), metal oxide (silica [14]–[18], titania [17], zirconia [19], magnetite [20]–[21] and barium titanate [22]), and metal sulphide (CdS [23], and Fe_2_S_3_ [21]) nanoparticles by using prokaryotic as well as eukaryotic organisms including bacteria [4]–[5], [9]–[10], [12], [21], [23], fungi [7]–[8], [14]–[17], [19]–[20], [22], and plants [6], [11], [13]. However, among various organisms studied until to date, prokaryotes remain the choice of organism for biological synthesis of nanomaterials [4]–[5], [9]–[10], [12], [21], [23]. This is predominantly because prokaryotes offer well-defined advantages over eukaryotic organisms such as easy handling, ease of downstream processing and ease of genetic manipulation. However, the full potential of prokaryotic organisms for biological synthesis of nanoparticles can only be realized when plausible biochemical mechanism of nanoparticle synthesis is clearly understood. Among synthesis of different nanoparticles by various microorganisms, bacterial synthesis of silver nanoparticles (AgNPs) is particularly attractive from microbiology perspective due to existence of well-known silver resistance machinery in few silver resistant bacterial species, thus making their study significantly important for biomedical applications [10]. Moreover, silver nanoparticles have remained an attractive choice of nanomaterial because of their ability of encompassing broad application area from electronics to medicine to food technology [3]–[4], [24]–[30].

Recently, in an attempt to understand the biomolecular mechanism of extracellular AgNPs synthesis, we demonstrated that *Morganella morganii* strain RP-42 isolate [10] was capable of synthesizing AgNPs extracellularly, and explored the phenotypic and genotypic characters of putative silver resistant machinery in *Morganella* sp. RP-42 [10]. It, however, remains an established fact that microbial physiology tends to evolve rapidly to the environmental changes to increase the chances of survival. To understand AgNPs synthesis by *Morganella* in a more clear way, it was found significantly important to evaluate whether the AgNPs synthesis was an adaptive physiology of *Morganella* sp. strain RP-42 as a result of environmental factors or it was independent to that [12]. In the present study, we aim to further strengthen the understanding of AgNPs synthesis in *Morganella*, and validate whether extracellular AgNPs synthesis in the presence of Ag^+^ ions is a genus-wide phenotype in *Morganella* spp. This has been achieved by performing a detailed time-dependent UV-visible absorption spectroscopy study with respect to AgNPs synthesis capability of all the known *Morganella* biogroups till date. We have also investigated the presence of gene homologue of putative gene of the silver binding protein (*silE*) from all strains of *Morganella* spp. that were tested for AgNPs synthesis. To achieve this, we have carried out a genus-wide characterization of AgNPs synthesis using all the members of genus *Morganella* isolated from different environments.

## Results and Discussion

On exposure to 5 mM colourless AgNO_3_ solutions, all *Morganella* biogroups formed dark brown coloured solutions within 20 h of reaction, except for *M. psychrotolerans* that formed greenish brown colloidal solution, indicating formation of extracellular AgNPs by all the biogroups. The color of the solutions did not significantly change from that point onward (except in intensity), even after continuing the reaction for up to 5 days. The AgNPs solutions remained stable for at least 8 weeks without any visible aggregation or precipitation. To understand the nature of nanoparticles, detailed physico-chemical characterization of extracellular AgNPs formed by all *Morganella* strains was carried out using UV-Vis absorbance spectroscopy, transmission electron microscopy (TEM), and X-ray diffraction (XRD) studies as described in the materials and methods section [10].

**Figure 1:**
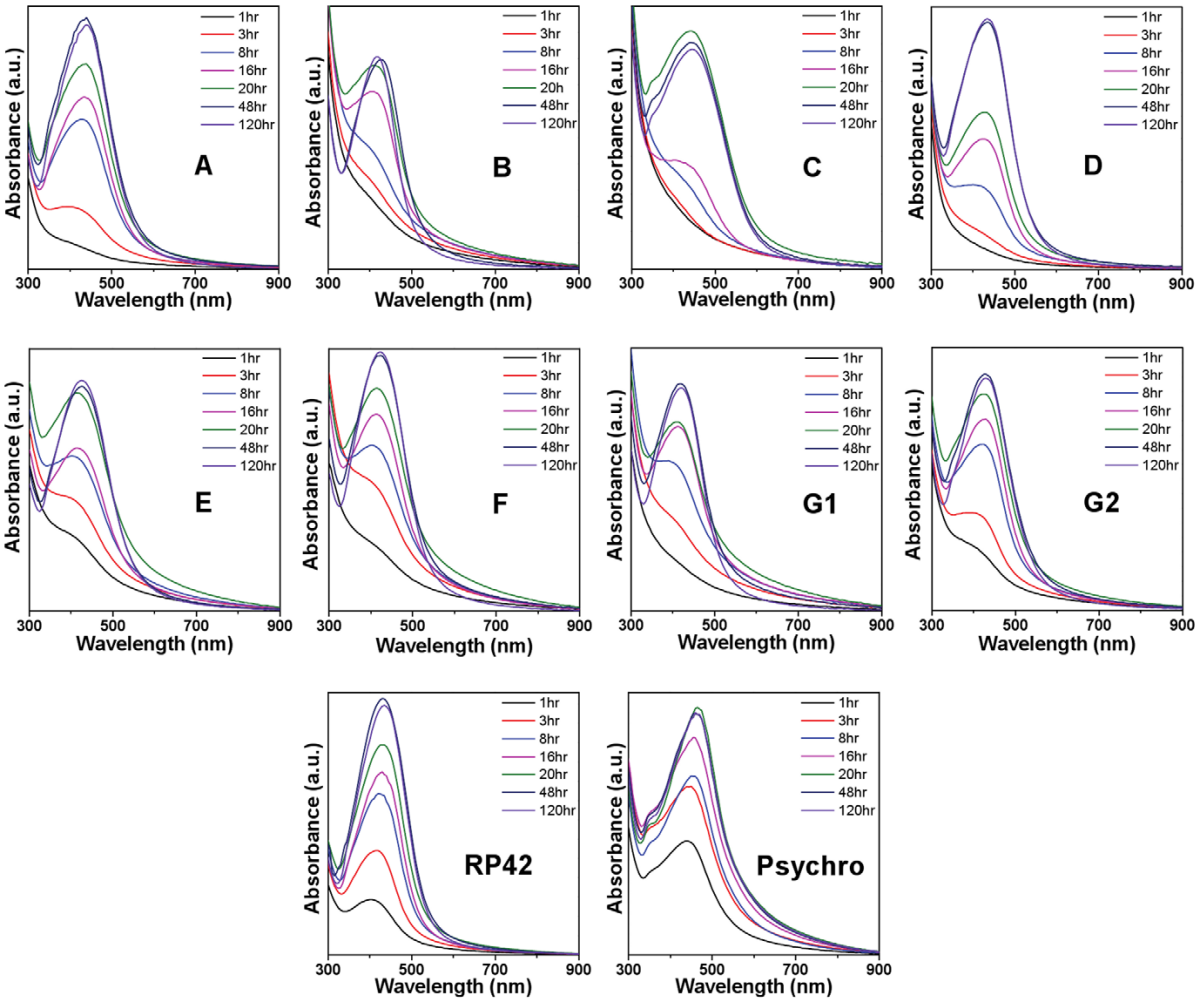
UV-vis spectra of culture supernatants from different biogroups of *Morganella* showing the extracellular synthesis of AgNPs in a time-dependent manner.

**Figure 2:**
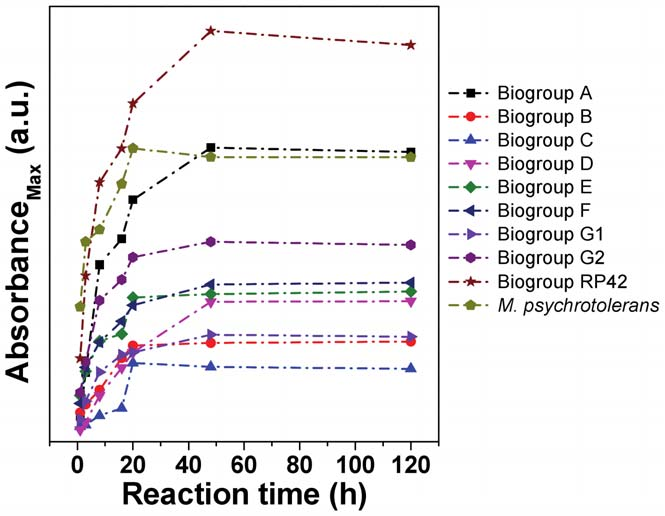
Comparative production of AgNPs by different biogroups of *Morganella* represented in terms of maximum absorbance SPR intensities of AgNPs plotted against biosynthesis reaction time.

Figure 1 shows the time-dependent UV-vis absorbance spectra of colloidal solutions obtained after reaction of all *Morganella* biogroups with 5 mM AgNO_3_ for 1, 3, 8, 16, 20, 48 and 120 h. The presence of a characteristic Ag surface plasmon resonance (SPR) between 400 and 500 nm is clearly evident in all the samples, thus confirming the formation of extracellular AgNPs by all *Morganella* biogroups [10]. The differences in the position of absorbance maxima of SPR features of AgNPs synthesized by different biogroups is notable, which is most likely due to the difference in the size and/or shape of Ag nanocrystals synthesized by these biogroups [12], [31,]. It is also interesting to note that most of the *Morganella* biogroups started synthesizing AgNPs as early as within 1 h of reaction and the yield of AgNPs by different biogroups increased as the reaction progresses over a period of time. However, the amount of AgNPs produced by different biogroups reached to a saturation state somewhere between 20 h and 120 h of reaction, which varied from one biogroup to another. This suggests that although all *Morganella* biogroups have the capability to reduce Ag^+^ ions to form AgNPs (Ag^0^), the rate of AgNPs formation may vary among them. To compare the rate of AgNPs formation by different biogroups, the maximum absorbance intensity (A_max_) of the Ag SPR feature of different biogroups was plotted with respect to different time points of the biosynthesis reaction (Figure 2). It is clearly evident from Figure 2 that different biogroups indeed followed different reaction kinetics in terms of AgNPs formation, among which *M. psychrotolerans* showed the fastest activity towards AgNPs biosynthesis, followed by *M. morganii* strain RP-42 (compare absorbance intensities at 1 h). However, after 8 h of reaction, AgNPs production by *M. morganii* strain RP-42 superseded that from *M. psychrotolerans*, thus RP-42 strain showing largest overall AgNPs production capability within 120 h time frame. In a control experiment, SPR signatures corresponding to Ag nanoparticles were found absent in the media control wherein no bacteria were inoculated, thus ruling out the possibility of direct role of media components on AgNPs synthesis, and affirming that AgNPs synthesis resulted as a whole function of extracellular micro-environment created by different strains of *Morganella*.

It is also interesting to note that AgNPs production by most of the *Morganella* biogroups (except biogroups RP-42 and A) reached to a saturation state within 20 h of reaction, after which no further increase in AgNPs synthesis was observed. Therefore, in this study, although UV-Vis analysis was performed up to 5 days to follow the reaction kinetic, we performed TEM and XRD analysis on AgNPs obtained after 20 h of reaction. In our opinion, 20 h time point provides a better representation than 120 h time point for comparison between AgNPs synthesized by different biogroups, predominantly because at 20 h time point AgNPs biosynthesis is in its log (growth) phase, which enables to capture the state of as-formed particles, rather than a possibility of their further modification while AgNPs stay in the bacterial growth media up to 120 h. It should also be noted that when we previously performed a detailed precursor concentration-dependent experiment on *M. morganii* strain RP-42, the rate of AgNPs formation was found to be maximum at 5 mM AgNO_3_ concentration, and was reduced by increasing the precursor concentration [10]. This motivated us to perform all the experiments reported in the current study at 5 mM AgNO_3_ concentration. However, considering the differences in the rate of AgNPs biosynthesis by different *Morganella* biogroups, it is likely that optimal precursor concentration for maximum AgNPs synthesis rate may vary from one biogroup to another. This will require separate detailed investigations concerning each of the biogroups in the future, wherein influence of various parameters such as precursor concentration, solution pH, reaction time and temperature should be studied in detail to obtain more insights about each system.

**Figure 3:**
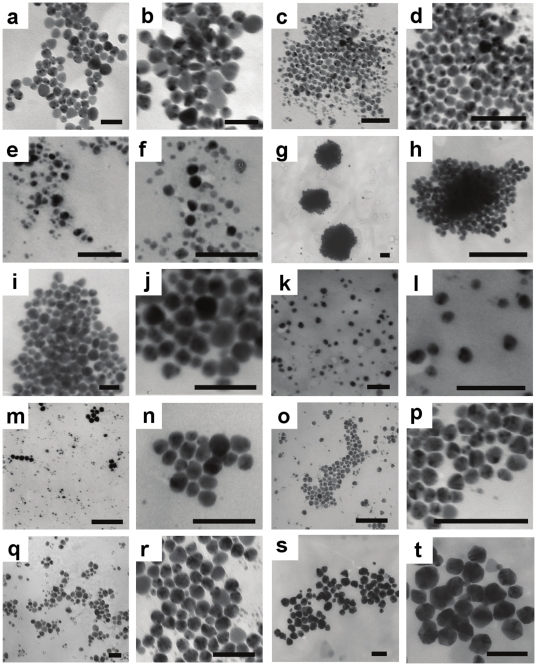
Transmission electron microscopy (TEM) images of extracellular AgNPs formed by different biogroups of *Morganella*. Biogroup A (panels a and b), biogroup B (panels c and d), biogroup C (panels e and f), biogroup D (panels g and h), biogroup E (panels i and j), biogroup F (panels k and l), biogroup G1 (panels m and n), biogroup G2 (panels o and p), *M. morganii* strain RP-42 (panels q and r), and *M. psychrotolerans* (panels s and t). Scale bar in each panel corresponds to 100 nm.

To understand the morphology of AgNPs formed by different biogroups of *Morganella*, the TEM analysis of AgNPs synthesized by all biogroups of *Morganella* was performed after 20 h of biosynthesis (Figure 3). It is evident from TEM images that AgNPs formed by all biogroups were quasi-spherical in shape, ranging 10–50 nm in diameter (Figure 3a–r). The particle size distribution of AgNPs formed by different *Morganella* biogroups was assessed using TEM micrographs for at least 200 particles in each biogroup, which revealed that the average particle diameters with the standard error of mean of quasi-spherical AgNPs synthesized by different biogroups were 39.9±1.1 nm (A), 19±1 nm (B), 12.3±0.7 nm (C), 10.2±0.2 nm (D), 32.9±1.3 nm (E), 15.1±0.9 nm (F), 8.3±1.1 nm (G1), 14.8±0.8 nm (G2), 32.8±1 nm (RP-42), and 46.3±1.2 nm (*M. psychrotolerans*).

**Figure 4:**
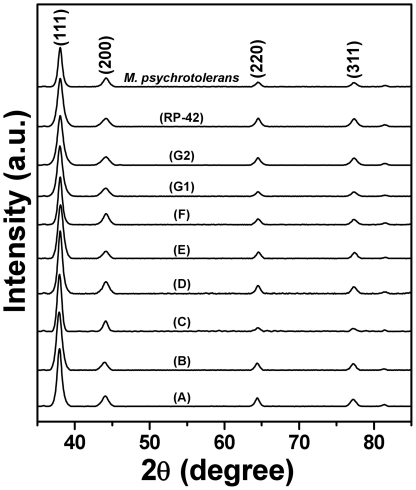
X-ray diffraction (XRD) patterns of extracellular AgNPs formed by different biogroups of *Morganella*. Each XRD pattern has been labelled with respective biogroup, and the Bragg reflections corresponding to (111), (200), (220), and (311) planes have been indicated that are characteristic of crystalline silver.

**Figure 5:**
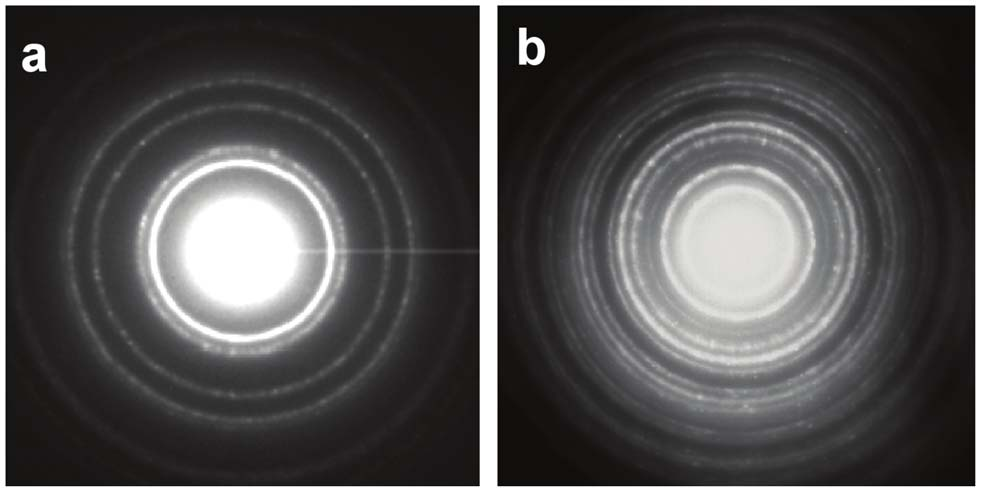
Selected area electron diffraction (SAED) patterns obtained from AgNPs formed by (A) *M. morganii* strain RP-42, and (B) *M. psychrotolerans*.

The crystallography of AgNPs formed by different biogroups of *Morganella* after 20 h of reaction was investigated by XRD. As is evident from XRD patterns in Figure 4, extracellular AgNPs synthesized by all the biogroups are highly crystalline in nature, that could be perfectly indexed to the {111}, {200}, {220} and {311} Bragg reflections of the face centered cubic (fcc) form of crystalline silver [12], [24], [31]–[32]. XRD analysis thus provided a clear indication of formation of high quality crystalline AgNPs using a *Morganella* mediated biosynthesis process by all its type strains. Additionally, the crystallinity of AgNPs was further confirmed by performing SAED analysis of AgNPs formed by *M. morganii* strain RP-42 and *M. psychrotolerans* during TEM imaging (Figure 5). The SAED patterns from both the samples revealed well-defined diffraction spots in the form of rings, which are indicative of polycrystalline silver.

The UV-vis, TEM, XRD and SAED results presented in this study clearly demonstrate that formation of AgNPs is a genus-wide characteristic phenotype of all reported type strains of *Morganella* to date. Further experiments were performed to explore whether AgNPs formation is a characteristic phenotype restricted to genus *Morganella*, or whether other taxonomically related genera of Enterobacteriaceae family also show this feature. To obtain this insight, when comparative analysis of AgNPs synthesis using laboratory strains of *Escherichia coli*, *Salmonella typhimurium*, *Kelebisella pneumoniae* and *Serratia marcescens* was performed in the presence of 5 mM AgNO_3_, no AgNPs formation was observed in any of these closely related organisms. Additionally, distant taxonomic relatives of *Morganella* such as *Firmicutes* and *Actinobacter* did not lead to formation of detectable AgNPs in solutions. This strongly suggests that AgNPs synthesis in the presence of Ag^+^ ions is a phenotypic character that is uniquely associated with genus *Morganella*.

**Figure 6:**
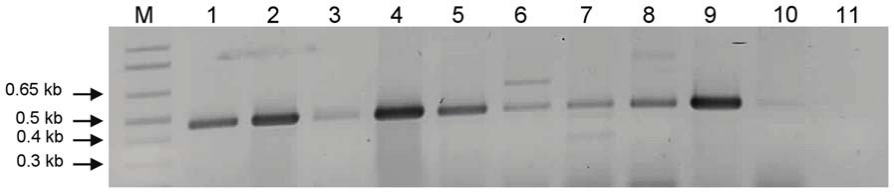
1% Ethidium bromide stained agarose gel shows PCR products of *silE* gene homologue from all biogroups/type strains of *Morganella* including *M. morganii* RP-42. (1) biogroup A, (2) biogroup B, (3) biogroup C, (4) biogroup D, (5) biogroup E, (6) biogroup F, (7) biogroup G1, (8) biogroup G2, (9) *M. morganii* RP-42, (10) *M. psychrotolerans*, (11) negative DNA control, (M) molecular weight DNA marker.

It has been previously established that in silver resistant bacteria, silver resistance mechanism involves the gene (SilE) which encodes a periplasmic silver binding protein (silE). This macromolecule plays a major role in highly specific uptake of Ag^+^ ions from surrounding environment by providing histidine sites as primary candidate for Ag^+^ ions binding [33]. Similarly, in our previous study, we established that silver resistance machinery in *Morganella morganii* RP-42 is associated with AgNPs synthesis capability of this particular strain [10]. Since, in the current study, all reported strains of genus *Morganella* were found to exhibit phenotype of AgNPs synthesis in the presence of Ag^+^ ions, we found it important to associate the presence of silver resistance (SilE gene) in all these strains with their AgNPs synthesis capability [10]. Therefore, to determine whether all strains of *Morganella* spp. exhibit silver resistance, further efforts were made to identify gene homologue of SilE in all the members of genus *Morganella*. As can be seen from Figure 6, all *Morganella* strains showed the presence of SilE gene homologue. This observation further strengthens the probable role of silver resistance genes and gene products in AgNPs synthesis in *Morganella*. Detailed investigation of functional role of silver resistance genes in AgNPs synthesis is currently being pursued.

In the present study, we have demonstrated that the phenotype of extracellular AgNPs synthesis in *Morganella* is not just restricted to an isolate pertaining to one environment, but it is indeed a unique biochemical character associated with all the members of this genus isolated from different environment [34]–[37]. This clearly establishes that AgNPs synthesis by genus *Morganella* is a phenotype independent of environmental influence. Although AgNPs synthesis has been previously reported by other microorganisms, this is for the first time that extracellular synthesis of AgNPs by all the members a particular Genus (*Morganella*) has been established, and their AgNPs synthesis capability has been followed in a time-dependent manner. The observation that members of other genera of the same *Enterobacteriacea* family are incapable of AgNPs synthesis, establishes AgNPs synthesis as a unique phenotypic character of genus *Morganella*. These observations might, in future, not only provide a complementary tool for easy detection and purification of *Morganella* in the presence of other members of *Enterobacteriaceae* family, but might also establish new evolutionary links between different microorganisms by comparing their metal ion reducing capabilities.

## Materials and Methods

### Growth and identification of *Morganella* strains

The genus *Morganella* comprises of two species viz. *Morganella morganii* with two sub-species *morganii* and *sibonii*, and *Morganella psychrotolerans*. On the basis of biochemical and taxonomic profiling, two sub-species *morganii* and *sibonii* have been further divided into a total of eight biogroups (Table 1) [34]–[37]. All strains of *Morganella* were routinely maintained on LB agar slants and preserved at −80°C. The identity of all strains was confirmed by 16S rRNA gene sequencing as shown earlier [10], and sequences were submitted to GenBank with accession numbers HM122047, HM122048, HM122049, HM122050, HM122051, HM122052, HM122053, HM122054 and HM122055.

**Table 1:** List of existing members of genus *Morganella*.

S. No.	Species	Sub-species	Biogroup	Strain no.
1	*Morganella morganii*	*morganii*	A	ATCC 25830
2	*Morganella morganii*	*morganii*	B	CDC 1939-76
3	*Morganella morganii*	*morganii*	C	CDC 1427-73
4	*Morganella morganii*	*morganii*	D	CDC 2866-78
5	*Morganella morganii*	*sibonii*	E	CDC 8293-1
6	*Morganella morganii*	*sibonii*	F	CDC 13-82
7	*Morganella morganii*	*sibonii*	G1	CDC 3522-75
8	*Morganella morganii*	*sibonii*	G2	CDC 8246-91
9	*Morganella psychrotolerans* U2/3	-	-	LMG 23374 = DSM 17886
10	*Morganella morganii*	*morganii*	RP-42	Reference strain for AgNPs synthesis

### Extracellular biosynthesis of silver nanoparticles by *Morganella* spp. and nanoparticles characterization

All reported type strains of *Morganella* were used for AgNPs synthesis in the present study (Table 1). All strains of *Morganella* were initially grown at 37°C for 24 h in a 500-mL Erlenmeyer flask that contained LB broth (100 mL) without added NaCl in a shaker incubator set at 200 rpm, as reported previously for *M. morganii* strain RP-42 [10], except for *M. psychrotolerans*, wherein the bacteria were grown at 20°C, while maintaining all other synthesis conditions similar to those for other *Morganella* strains. For *M. psychrotolerans*, 20°C was used as the growth temperature, because this being a psychrotolerant species, 15–20°C is its optimum growth temperature and *M. psychrotolerans* could not be grown successfully at 37°C even after 5 days of incubation [12]. For all other *Morganella* biogroups, the optimum growth temperature being 37°C, they were grown at 37°C. Following bacterial growth, all the culture suspensions were incubated with aqueous 5 mM solutions of AgNO_3_ at 37°C in a shaker incubator at 200 rpm in the dark, and the reactions were carried out in a time-dependent manner for up to 120 h (5 days). The *M. morganii* strain RP-42 that was earlier reported by us to synthesize extracellular AgNPs was considered as a positive control in all the experiments [10]. The extracellular synthesis of AgNPs was initially detected by visual inspection of the culture flask for a change in color of culture medium from clear light-yellow to brown/green. Extracellular AgNPs were separated from bacterial cells by centrifuging aliquots of culture supernatants (1.5 mL) at 3000 rpm for 6 min at 25°C. The supernatants thus obtained were clear brown/green homogenous suspensions of AgNPs, which were analyzed in a time-dependent fashion using UV-vis spectroscopy (Cary 50 Bio-spectrophotometer) at a spectral resolution of 2 nm, transmission electron microscopy (Jeol 1010 TEM) at an accelerating voltage of 100 keV, selected area electron diffraction (SAED) coupled with TEM instrument, and X-ray diffraction (XRD - Bruker AXS D8 Discover) using Cu K_α_ radiation and a General Area Detector Diffraction System (GADDS). For UV-vis analysis, the AgNPs suspensions were diluted 10 times using MilliQ deionized water at every time point and UV-vis spectra were obtained. For TEM analysis, AgNPs samples obtained after 20 h of reaction were prepared by drop casting the colloidal suspensions of AgNPs onto carbon-coated Cu grids followed by drying under air for 24 hours. For XRD analysis, the samples were prepared by precipitating AgNPs obtained after 20 h of biosynthesis at 10,000 rpm for 15 min, followed by three washings with MilliQ deionised water, and drop casting the samples onto a glass substrate.
